# Therapeutic applications of nanobots and nanocarriers in cancer treatment

**DOI:** 10.1007/s44211-025-00799-5

**Published:** 2025-06-04

**Authors:** Zaitoon Khan, Nimra Khan, Mithra Geetha, Reyhanath Pilakka Veettil, Deepak Mahadev Kasote, Anwarul Hasan, Kishor Kumar Sadasivuni

**Affiliations:** 1 Center for Advanced Materials, Qatar University, Doha, Qatar; 2 Agricultural Research Station, Qatar University, Doha, Qatar; 3 Department of Mechanical and Industrial Engineering, Qatar University, Doha, Qatar

**Keywords:** Cancer treatment, Nanobots, Nanocarriers, Nano-formulation, Drug delivery

## Abstract

Cancer remains a pressing global health concern, burdening individuals, families, and healthcare systems worldwide, mainly because of the limited availability of therapeutic options. These days, nanotechnology is revolutionizing cancer research by offering promising drug delivery systems. Among these, nanobots and nanocarriers are key nanotechnology components, offering groundbreaking strategies in cancer therapy by delivering drugs, enhancing diagnostics, and preventing diseases at molecular levels. Nanobots and nanocarriers, engineered at sizes ranging from 1 to 100 nm, demonstrate biocompatibility with cellular systems, offer precise drug delivery, and reduce side effects compared to conventional therapies such as chemotherapy and surgery. Research efforts now focus on transitioning nanobots and nanocarriers from laboratories to clinics, aiming for precision medicine, targeted drug delivery, enhanced therapy efficacy, and minimal side effects. This review aims to highlight the potential of nanobots and nanocarriers in cancer treatment and provide insights into their role in paving the way for clinical applications and improving outcomes in cancer therapy.

**Graphical abstract:**

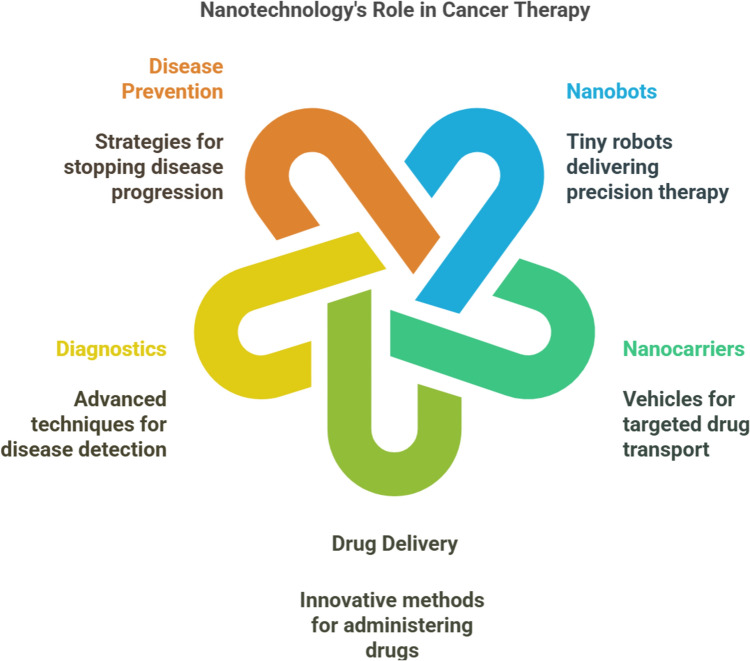

## Introduction

Cancer is one of the leading causes of death worldwide these days. The word “cancer” is derived from the Greek word “karkinos”, which means tumor [1]. The condition was called cancer in ancient times because an advanced cancer was thought to resemble a crab, with “claws” reaching out into surrounding tissues. Hence, the regulation and management of cell damage are crucial in maintaining the integrity of tissues, and these processes are collectively termed tumor suppression mechanisms [2]. As the incidence of cancer continues to rise due to changing environmental factors and lifestyles, there is an increasing need to develop diverse therapeutic strategies to combat it.

Ancient transcripts from Egyptian and Greek civilizations provide insights into historical surgical procedures for removing cancerous tissues or tumors along with natural medicines [3]. Notably, nitrogen mustard, developed during World War II, marked an early milestone in chemotherapy. Radium therapy, involving the use of radioactive radium to shrink tumors, was another prevalent treatment, eventually replaced by more precise radiation therapies [4].

Among the existing therapies, chemotherapy, a well-established cancer treatment, continues to be a recommended approach. However, combinatorial approaches, such as surgery and chemotherapy interventions, are collectively employed in cancer treatment [5]. Recently, hormonal therapy, which involves manipulating hormones to treat hormone-sensitive cancers, has effectively been use. In this treatment, estrogen-blocking drugs are administered to treat breast cancer. In the late nineteenth and early twentieth centuries, researchers began exploring immunological methods as a potential treatment for cancer, laying the foundation for modern immunotherapies such as monoclonal antibodies and interferon-based therapies [6]. However, the problem with existing cancer treatments, such as chemotherapy and radiation therapy, is that they have considerable side effects, mainly due to their inverse action on healthy cells and burst release. Therefore, it is necessary to develop an organized structure that could enable a sustained release of drugs, overcoming the first-pass metabolism and drug side effects.

In recent years, the sustained release of the drug has been achieved through nanostructures that could deliver the drug at the desired site with reduced side effects and potent pharmacological response [7]. However, cancer cells develop a defense mechanism through overexpression of efflux pumps, self-repairing capacity, altered targets, or increased drug metabolism [8]. This may affect the treatment process, highlighting the need for specialized nanocarriers (structures or systems, typically made of nanoparticles, that are designed to carry and deliver substances (e.g., drugs, genes, or other bioactive molecules) to targeted cells or tissues. They are commonly used in medical applications to improve drug delivery efficiency by controlling the release rate and targeting specific locations within the body) to overcome all its defense mechanisms. Considering this, in recent times, nanocarriers and nanobots are small, autonomous, or semi-autonomous machines or robots designed to perform specific tasks at the nanoscale (typically in the range of 1–100 nm). They can be programmed or controlled to carry out tasks such as drug delivery, environmental monitoring, or surgery. The term is often used in the context of medical or biological applications and have emerged as promising tools for delivering cancer therapies due to their unique properties. These tiny structures can transport therapeutic agents such as peptides, nucleic acids, or chemotherapeutic drugs. Unlike conventional nanomedicine approaches to cancer treatment, which often overlook factors like patient genomic profile, sexual dimorphism, biological aging, commensal diversity, and intratumor genomic heterogeneity, nanocarriers and nanobots propel advancements toward precision oncology [9].

This article captured recent advancements in nanobots and nanocarrier technologies, highlighting their pivotal role in combating cancer. This mainly includes a rigorous analysis of existing literature about the intricate functionalities of nanobots, miniature devices engineered for precise tasks at the nanoscale, nanocarriers, and nano-sized vehicles tailored for targeted drug delivery. In addition, we have summarized details of diverse strategies employed to optimize the design and fabrication of these nanostructures, enhancing their biocompatibility, stability, and controllability, and highlighted the unique abilities of nanobots and nanocarriers in delivering therapeutic payloads directly to tumor sites, minimizing off-target effects, and overcome drug resistance. In addition, herein, we have discussed the synergistic interactions between nanotechnology and conventional cancer therapies, paving the way for enhanced treatment outcomes, addressed challenges, and proposed innovative strategies for advancing the field.

## Nanobots and nanocarriers

Nanotechnology combines chemistry, physics, materials science, and biology to combine the collective expertise needed to develop these novel technologies [10]. Nanobots are autonomous or semi-autonomous nanoscale devices capable of navigating biological environments, actively targeting cancer cells, and performing precise therapeutic functions such as drug delivery, imaging, and tumor ablation. In contrast, nanocarriers are passive or responsive nanoscale systems designed to encapsulate and transport therapeutic agents to tumor sites, improving drug bioavailability and minimizing systemic toxicity. It finds applications in various fields, such as medicine, cosmetics, and environmental and nutraceutical research [11]. Different forms of nanostructures, such as nanofibers, nanocomposites, nanoparticles, and nanotubes, effectively diagnose and treat various diseases [12]. These nanostructures are also employed as carrier molecules or transporting agents for vaccines, drugs, genes, proteins, and enzymes [13]. The peculiar quantum properties of these nanostructures also enable them to be applied in the agri-food industry [14]. Nanorobots (A more advanced form of nanobots, nanorobots are essentially self-replicating, programmable microscopic robots that can carry out complex tasks within a system (e.g., the human body). They are generally designed with capabilities for sensing, computation, and action. They often refer to highly specialized robotic structures with specific control systems) which are mainly nanodevices used to provide protection or treatment against human pathogens. They are designed to perform particular or sometimes tasks with precision at nanoscale dimensions of 1–100 nm. They are expected to work at atomic, molecular, and cellular levels in both medical and industrial fields. Advances in the areas of robotics, nanostructuring, medicine, bioinformatics, and computers can lead to the development of the nanorobot drug delivery system. Some of the examples are respirate nanorobots, microbivore nanorobots, surgical nanorobots, and cellular repair nanorobots. They have a diameter of about 0.5 to 3 microns and will be constructed out of parts with dimensions of 1–100 nm. The main element used in nanorobots is carbon because of its inertness and strength in the form of diamond and fullerene. They generally have an exterior passive diamond coating to avoid attack by the host immune system [15]. Techniques like scanning electron microscopy (SEM) and atomic force microscopy (AFM) are employed to understand the molecular structure of the nanoscaled devices.

Nanocarriers are colloidal nanoparticles that transport a therapeutic agent or other substances to a target site. The size of the nanocarriers lies between 1 and 100 nm (nm) in diameter. In contrast, the nanocarriers in the therapeutic application have to be less than 200 nm as the micro-capillaries of the body are 200 nm [16]. These nanocarriers provide good biocompatibility as they are inactive and generally regarded as a safe medium. These nanocarriers will have a long-term circulation period with the sustained release of drugs overcoming the endosome–lysosome mechanism. Modifying the physiochemical properties of nanocarriers, such as surface, composition, and shape, can enhance their activity with decreased secondary effects [17]. Thus, it creates a plethora of impacts in the field of drug delivery. Though a wide range of nanocarriers are developed, only a few possess a remarkable ability to transport the drug to the targeted site. Some unique features of nanocarriers include enhanced biodistribution and pharmacokinetics, enhanced stability, enhanced solubility, and reduced toxicity sustained and targeted drug delivery [18].

## Fabrication of nanobots and nanocarriers

Manufacturing nanobodies or nanomaterials derived from molecular components are highly challenging due to the field’s interdisciplinary nature. Various processing techniques are essential to integrate different functional materials into nanobots. These techniques involve the construction of tiny, layered structures with specific functionalities achieved through physical or chemical methods [19]. The development of functional nanobots for use in living organisms requires careful consideration of factors that could impact their effectiveness, such as degradation by internal enzymes, pH variations, and other external influences [20]. Nanobots are crafted by precisely manipulating molecules at the nanoscale, emphasizing the need for meticulous control and accuracy in their production [21] (Table 1).

**Table 1:** Comparison of nanobots and nanocarriers in cancer therapy

Feature	Nanobots	Nanocarriers	
Definition	Autonomous or semi-autonomous nanoscale devices designed for active targeting and therapeutic interventions	Passive or stimuli-responsive nanoscale systems designed to encapsulate and deliver drugs	[11]
Mechanism	Actively navigate and interact with biological environments	Rely on passive diffusion, enhanced permeability, and retention (EPR) effect or external stimuli	[12]
Functionality	Targeted drug delivery, real-time monitoring, tumor ablation, and minimally invasive surgery	Controlled drug release, improved bioavailability, and prolonged circulation time	[13]
Mobility	Self-propelled or externally guided	Lacks active mobility, relies on circulation and targeting ligands	[14]
Precision	High precision due to autonomous targeting	Moderate precision, often influenced by passive targeting mechanisms	[15]
Clinical application potential	Emerging, with ongoing research in autonomous nanomedical applications	More advanced clinical trials and approved formulations for cancer therapy	[15]

Nanobots are commonly produced using two main fabrication approaches: top-down and bottom-up. In the top-down method, nanobots are created by reducing bulk materials into smaller nano-sized particles through techniques like lithography and etching [22]. On the other hand, the bottom-up approach involves assembling molecules from small building blocks using processes such as self-assembly or directed assembly without external manipulation. This method is beneficial for creating organic nanorobots like DNA origami structures [23]. DNA nanobots are utilized to release specific strands after assembling them accordingly. FESEM and TEM images of nanostructures are provided to visualize the resulting assemblies.

**Fig. 1:**
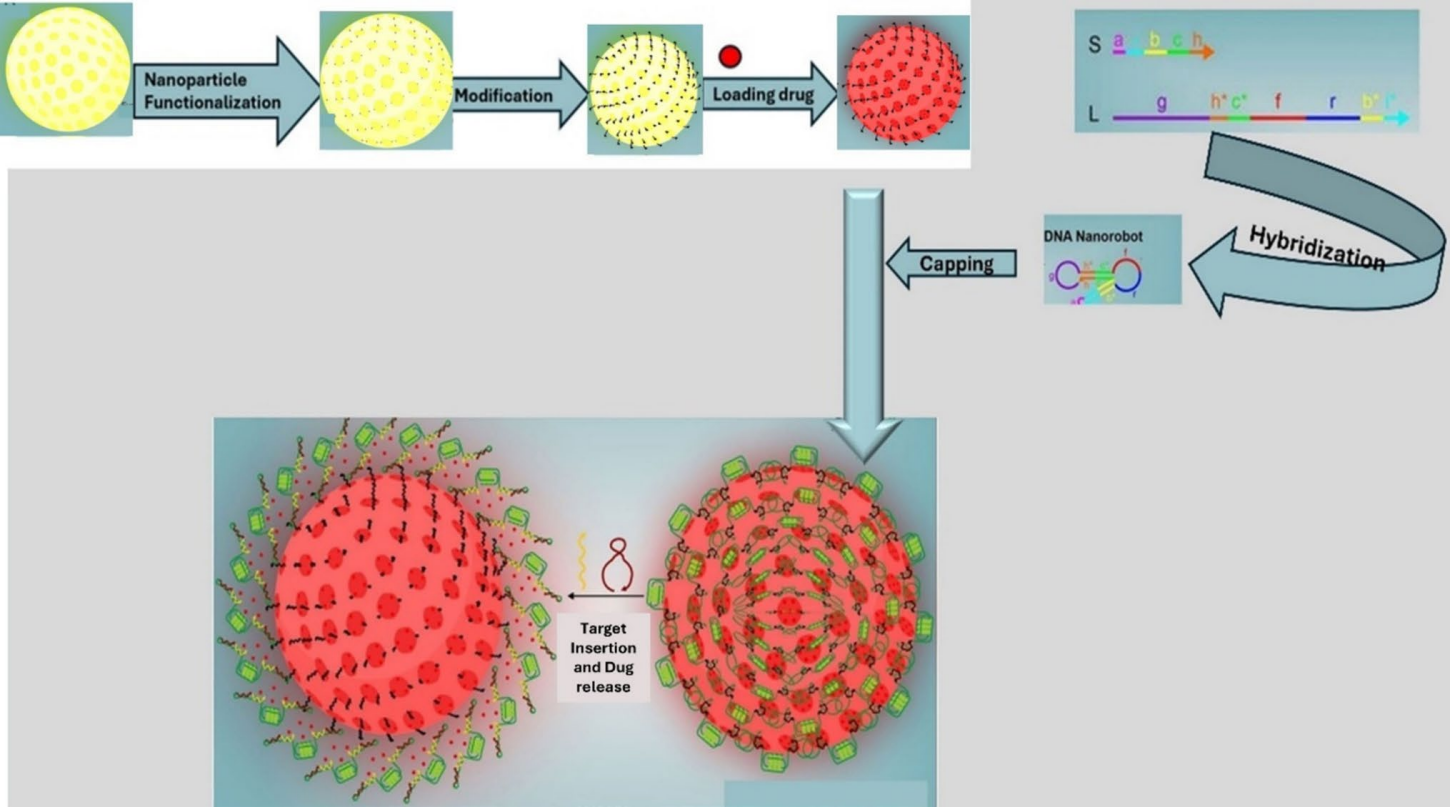
The DNA nanobot (S-L hybrid) is created by assembling the S and L strands, and the target strands are inserted into the DNA nanobot using an AND logic gate to release the L-T1-T2 hybrid and S strand [24]

Nanobots can also be synthesized through hybrid approaches, such as template-assisted assembly, which combines elements of both top-down and bottom-up methods using templates or scaffolds to guide nanoparticle assembly [23, 24]. This can then interact with specific cellular components, enabling precise delivery of therapeutic agents or the modulation of gene expression in targeted cells (Fig. 1). This innovative approach allows for enhanced specificity in treatment, minimizing off-target effects and improving overall therapeutic efficacy. Furthermore, the modular design of the DNA nanobot facilitates the incorporation of various payloads, allowing for versatile applications in gene therapy, cancer treatment, and regenerative medicine. For instance, Mirzaiebadizi and colleagues developed an intelligent DNA nanobot for detecting miRNA cancer biomarkers, utilizing molecular programming to create a logic-responsive hybrid nanostructure.

**Fig. 2:**
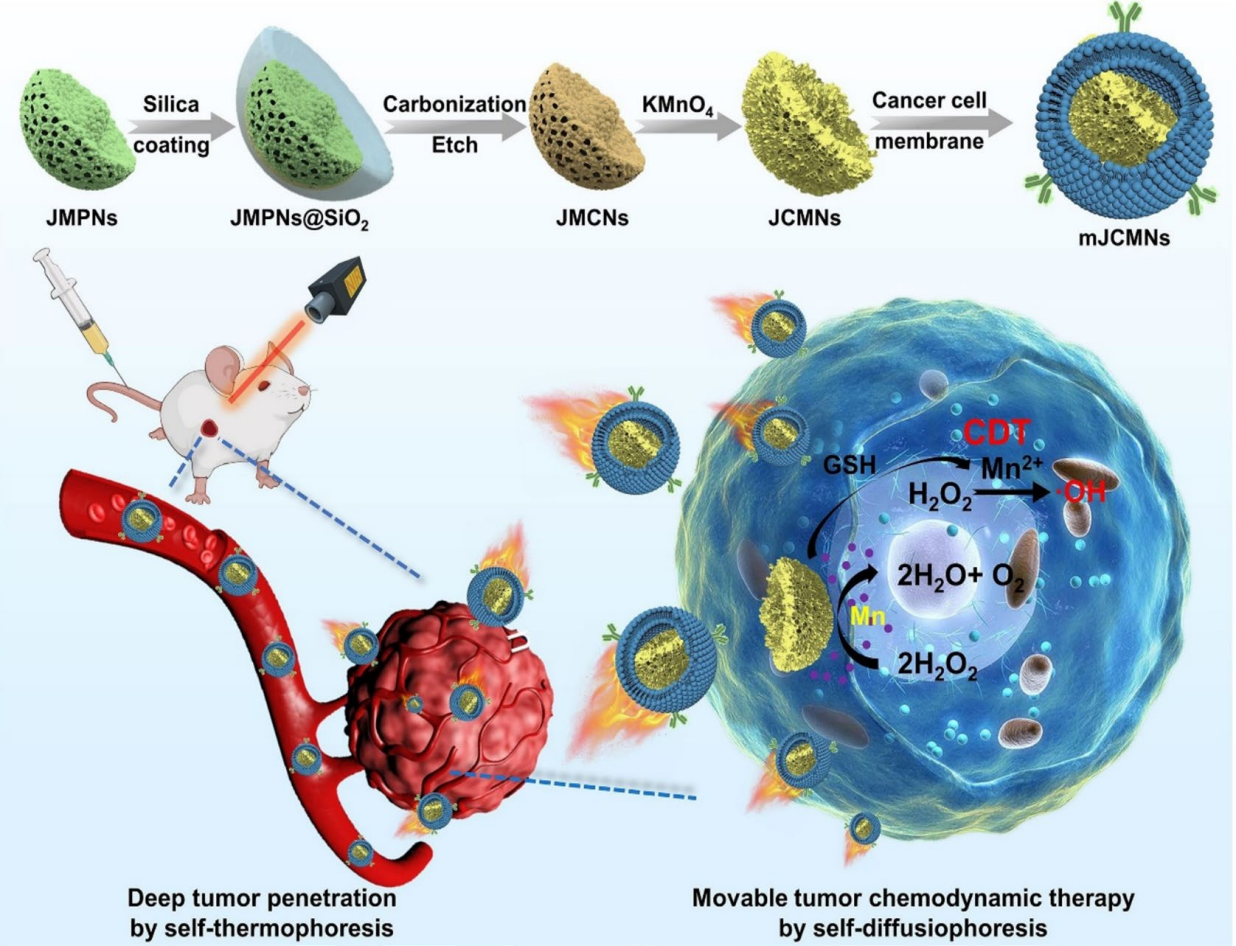
Illustration of fabricating mJCMNs and their improved CDT using NIR light and H_2_O_2_ dual propulsion [27]

In addition, nanobots have been fabricated by emulating naturally occurring nanobots in living systems, such as enzymes, organelles, DNA, and proteins. Biohybrid nanobots are emerging as promising platforms due to their biocompatible and biodegradable properties [25]. Various bioinspired models of nanobots have been developed (Fig. 2), including jellyfish-inspired nanobots for enhanced tumor penetration and improved chemodynamic therapy (CDT), as well as carbon/manganese nanomotors (JCMNs) designed for dual propulsion using hydrogen peroxide (H_2_O_2_) and near-infrared light [26].

On the other hand, nanocarriers have also been developed for the delivery of drugs to targeted sites, but the main difference between nanobots and nanocarriers is their power systems [28]. The emulsion/solvent evaporation technique fabricates nanocarriers. Nanoemulsions are emulsions with droplet sizes in the nanoscale range that are kinetically stabilized. These nanodroplets can include areas where polymerization or precipitation events can occur, creating particles and capsules that act as nanocarriers carrying drugs to cancer cells. It is a suitable fabrication method for hydrophobic and hydrophilic drugs, giving control over drug loading and nanoparticle size [29].

Another technique utilized for manufacturing nanocarriers is the layer-by-layer assembly method. This technique involves sequentially coating a template surface or nanoparticle core with alternating layers of polymers possessing opposing charges. Various types of bonds, such as covalent, hydrogen, and electrostatic, can be used in this process [30]. Gibson and colleagues, for example, developed a layer-by-layer assembled nanocarrier loaded with hyaluronic-decorated lipid nanoparticles (HA-LNP) to target Glioblastoma multiform (GBM) cells. Delivery of miR-181a via HA-LNP resulted in significant cellular death of U87 GBM cells in vitro and delayed tumor growth in an in vivo subcutaneous tumor model. Furthermore, some nanocarriers are created using microfluidic techniques, which involve manipulating fluids in microscale channels to generate nanoparticles. These microfluidic platforms offer precise particle size and morphology control through nanoprecipitation, emulsion/solvent evaporation, or self-assembly [31].

## Applications of nanobots and nanocarriers in cancer treatments

Nanotechnology emerged in the early 1980s, and questions arose regarding its potential applications for humanity. The scanning tunneling microscope (STM) invention by scientists Heinrich Rohrer and Gerd Binnig marked a significant milestone, enabling the observation and manipulation of individual atoms at the nanoscale [31]. This breakthrough paved the way for exploring the need for nanobots and nanocarriers, particularly in addressing the limitations, lack of specificity, and technical challenges associated with routine cancer therapies. Nanotechnology has since evolved as a promising avenue for cancer treatment, yielding miniature devices in nanobots and nanocarriers, which offer targeted and precise delivery of therapeutic agents [32].

**Fig. 3:**
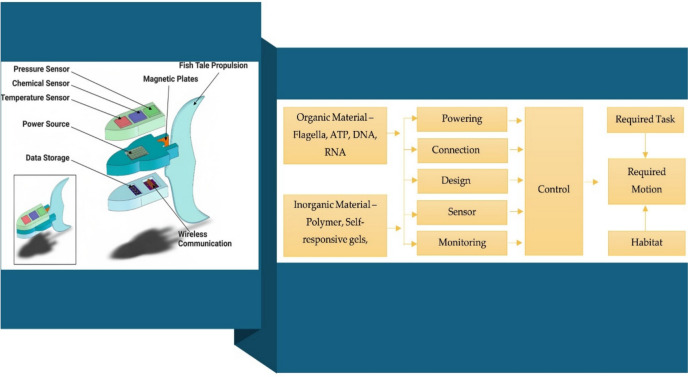
An illustrative overview of basic components of nanobots for cancer therapy

Compared to conventional cancer therapies, nanocarriers and nanobots offer several advantages. They demonstrate improved penetration into tumor tissues, surpassing obstacles that typically limit the effectiveness of traditional treatments. Nanocarriers excel in tumor penetration due to their high load-carrying capacity, facilitated by their elevated surface-to-volume ratio. In addition, current anticancer drugs, reliant on blood fluidics for tumor tissue penetration, benefit from the multidimensional structures of nanobots, as depicted in Fig. 3 [33]. Furthermore, nanocarriers and nanobots enhance therapeutic efficacy by delivering anticancer drugs directly into cancer cells, ensuring high drug concentrations at targeted sites through sophisticated systematic structures, as illustrated in Fig. 3. Nanocarriers and nanobots can be engineered to deliver drugs specifically to tumor tissues or adjacent malignant cells with precise control. This targeting can be activated by biological cues or external signals such as light or magnetic fields [34].

**Fig. 4:**
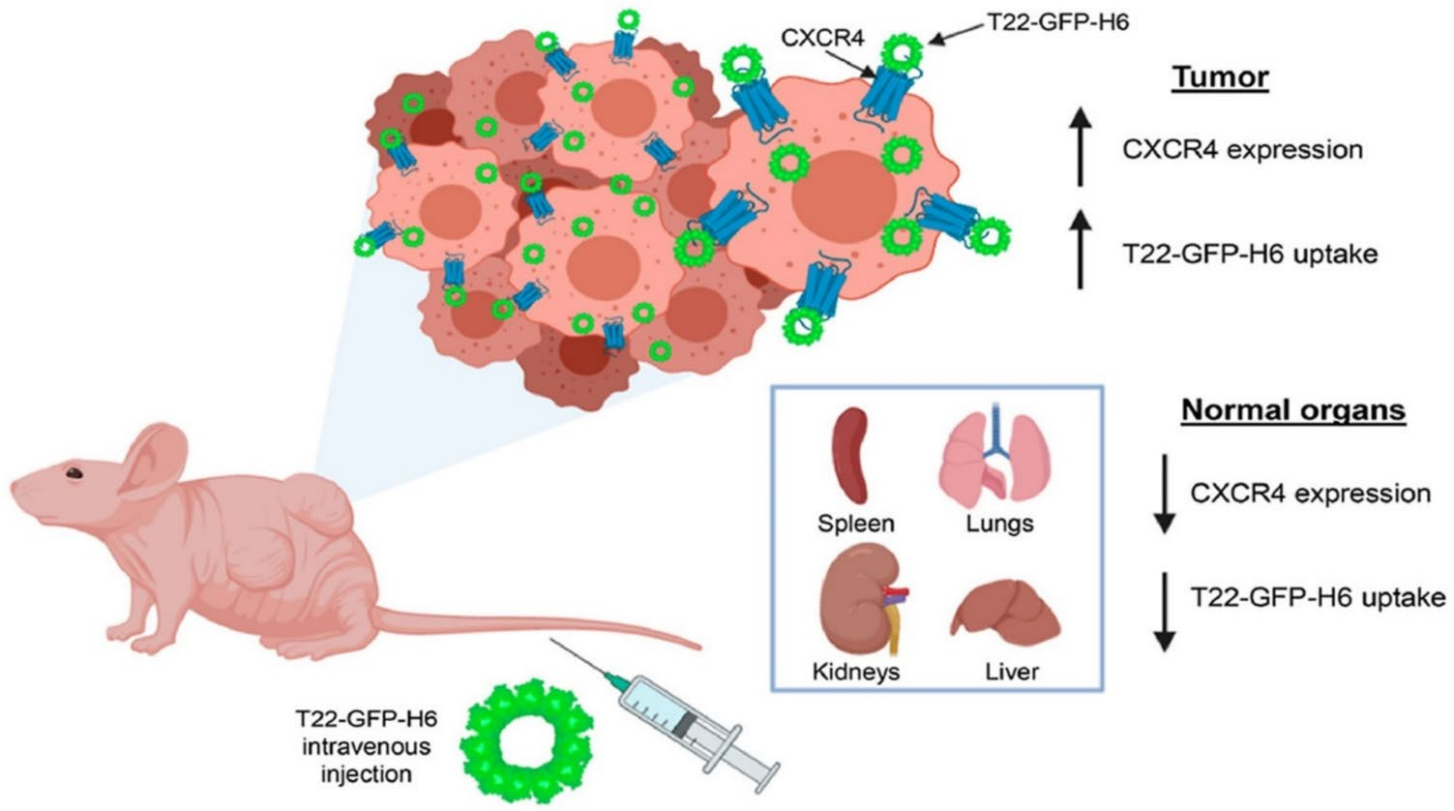
Deploying cytotoxic polypeptides specifically to CXCR4+ head and neck squamous cell carcinoma tumors by a self-assembling protein nanocarrier [36]

Lou and co-workers have developed a new CD44-targeting nanocarrier that enhances immune chemotherapy by inhibiting iRhom1. Blanco et al. studied a self-assembled biohybrid nanocarrier designed for delivering cytotoxic polypeptides to CXCR4+ neck squamous cell carcinoma tumors in a mouse model, as illustrated in Fig. 4. Furthermore, nanobots and nanocarriers play a crucial role in overcoming drug resistance in cancer cells by delivering a combination of drugs or evading resistance mechanisms. This enhances treatment efficacy and reduces side effects on cancerous tissues. Tailored treatment strategies, considering individual patient attributes, are a key feature of precision medicine that nanobots and nanocarriers can facilitate [35].

“Nanocarriers” typically denote engineered nanostructures explicitly designed for cancer treatment, such as drug transportation or pharmacokinetics within the body. These carriers facilitate transporting and delivering drugs or genetic material to targeted sites within the body [37]. They can enhance drug solubility, prevent payload degradation, and enable controlled release at designated locations [38]. Moreover, customized ligands with targeting capabilities are often added to the surfaces of nanocarriers [39]. By precisely delivering drugs to cancer cells, nanocarriers facilitate targeted damage to tumor tissues, improving treatment accuracy through active and passive targeting of metastatic tissues. Nanocarriers with enhanced biocompatibility can potentially reduce adverse effects on the body. Altering nanocarrier surfaces can also help evade the immune system. Modifying nanocarriers in size, surface charge, and composition can optimize their qualities for medication administration, ultimately enhancing the safety and efficacy of cancer therapies [40].

**Fig. 5:**
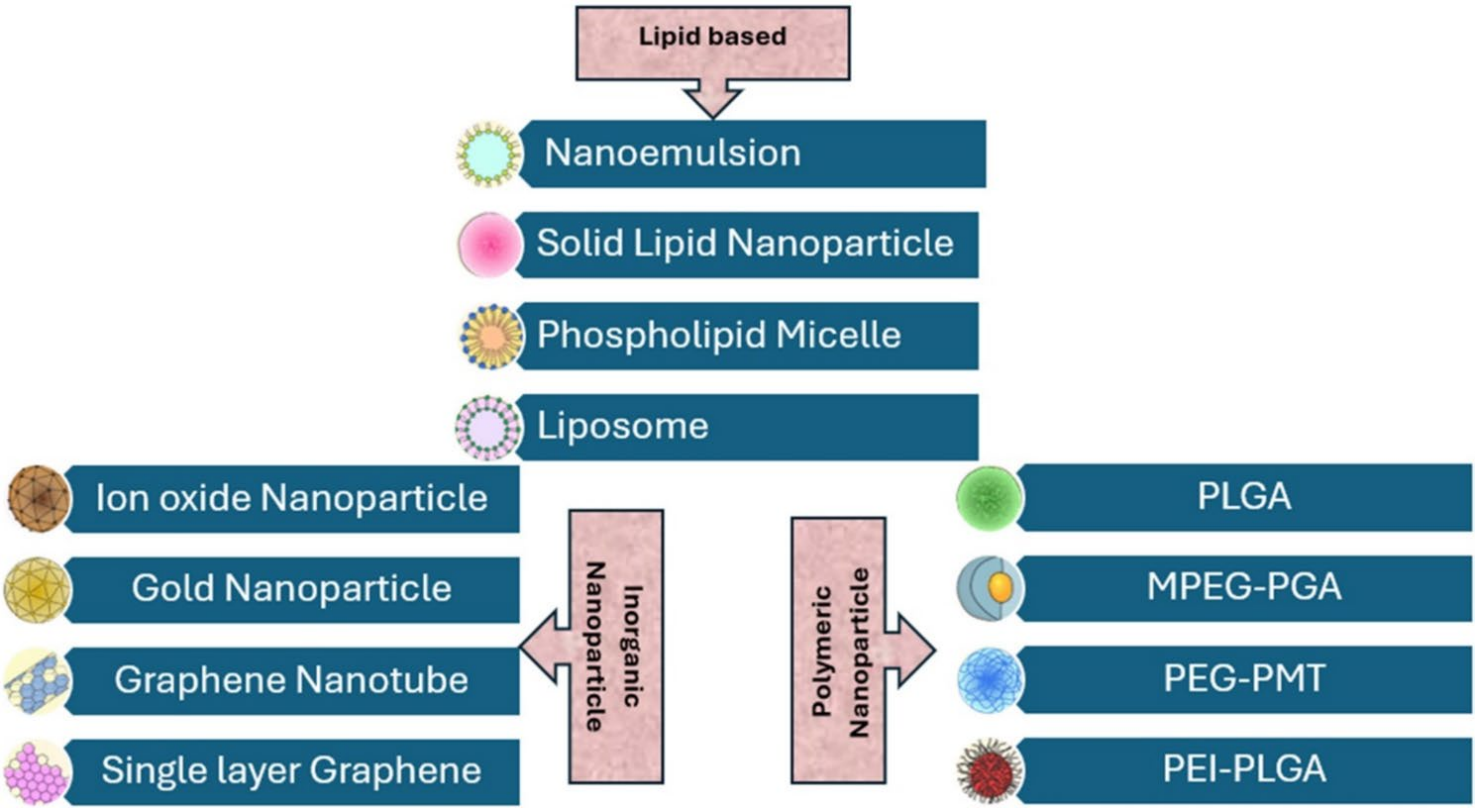
Different types of developed nanocarriers

Nanocarriers represent a significant advancement in cancer therapy, providing versatile platforms for encapsulating various medicinal substances, such as hydrophobic drugs, nucleic acids, and imaging agents. Their versatility allows for tailoring in different cancer treatment modalities, making them adaptable and effective tools in cancer therapy [40]. These nanocarriers deliver therapeutic payloads, especially drugs, in a targeted and controlled manner, enhancing the efficacy of treatments [41]. The three main types of nanocarriers—lipid-based, inorganic, and polymeric—differ in their physical and biochemical properties, offering unique advantages in cancer therapy (Fig. 5). Lipid-based nanocarriers comprise lipids that mimic cell membranes, including liposomes and solid lipid nanoparticles. Their amphiphilic nature allows for the encapsulation of both hydrophobic and hydrophilic drugs. Lipid-based nanocarriers are biocompatible, biodegradable, and can actively incorporate surface modifications to target cancer cells. They are handy for delivering hydrophobic drugs and nucleic acids with minimal toxicity [42, 43]. Inorganic nanocarriers, such as gold nanoparticles, silica nanoparticles, and quantum dots, are known for their stability, ease of functionalization, and ability to carry various therapeutic agents, including drugs, proteins, and imaging. These carriers often have excellent surface-area-to-volume ratios, making them efficient for drug loading and targeted delivery. Inorganic carriers can also be engineered to respond to specific stimuli, such as magnetic fields or pH changes in the tumor microenvironment [44].

Polymeric nanocarriers, such as PLGA (poly(lactic-co-glycolic acid)) and PEGylated nanoparticles, are biodegradable and offer controlled release of therapeutic agents. These carriers can be designed for sustained drug release, enhancing treatment effectiveness. Polymeric nanocarriers can be tailored to improve biocompatibility and stability, and their surface properties can be modified for targeted delivery to specific cancer cells. They are particularly effective in delivering hydrophilic and hydrophobic drugs [45, 46]. Nanocarriers, in general, are used for controlled and targeted drug delivery. Lipid-based carriers are ideal for hydrophobic drug delivery and gene therapy, inorganic nanocarriers excel in imaging and drug delivery applications due to their unique optical and magnetic properties, and polymeric carriers are widely used for sustained drug release and targeting specific cancer types. The use of these nanocarriers in combination with targeting strategies, such as ligand–receptor interactions or pH-sensitive release mechanisms, ensures that therapeutic payloads are explicitly delivered to cancer cells, improving treatment efficacy while minimizing systemic toxicity [44]. This dynamic landscape of nanocarrier development underscores their significance as promising platforms for advancing cancer treatments.

In addition to drug delivery, nanorobots show great promise in cancer diagnostics and monitoring. These tiny machines can be designed for in vivo imaging and biosensing, enabling early detection of cancerous growths and real-time monitoring of treatment response. Nanorobots can also detect circulating tumor cells in the bloodstream, potentially allowing for earlier diagnosis and more effective tracking of metastatic spread. Furthermore, they offer the capability for molecular profiling of tumors, providing valuable information for personalized treatment strategies [45].

Beyond drug delivery and diagnostics, nanorobots can actively participate in cancer treatment through various therapeutic interventions. Photothermal and photodynamic therapies utilize nanorobots to generate localized heat or reactive oxygen species upon light activation, selectively destroying cancer cells. Nanorobots can also enhance radiotherapy’s effectiveness by increasing tumor cells’ sensitivity to radiation. In gene therapy, nanorobots are efficient vectors for delivering therapeutic genes or RNA interference molecules to cancer cells. In addition, nanorobots show potential in revolutionizing cancer surgery, enabling minimally invasive tumor removal, precise identification of tumor margins, and even microsurgery on blood vessels supplying tumors. These surgical applications, coupled with the ability to monitor post-surgical sites and deliver localized treatments, highlight the versatility of nanorobots in comprehensive cancer care [46].

In addition to drug delivery, nanorobots (nanobots) show great promise in cancer diagnostics and monitoring. These tiny machines can be designed for in vivo imaging and biosensing, enabling early detection of cancerous growths and real-time monitoring of treatment response. Nanorobots can also detect circulating tumor cells in the bloodstream, potentially allowing for earlier diagnosis and more effective tracking of metastatic spread. Furthermore, they offer the capability for molecular profiling of tumors, providing valuable information for personalized treatment strategies [45]. For instance, Li et al. developed DNA origami-based nanobots capable of autonomously seeking out tumors and delivering thrombin to tumor-associated blood vessels, causing selective blood clotting within the tumor vasculature and leading to necrosis of cancer cells without harming healthy tissues [47]. These nanobots demonstrated effective tumor growth inhibition and high safety profiles in preclinical mouse models. Similarly, Wang et al. reported magnetically controlled nanobots for precision tumor targeting. These nanobots, composed of biocompatible materials, could navigate through biological fluids and actively home to tumor tissues under external magnetic guidance, facilitating localized drug delivery and minimizing systemic toxicity [48]. Gupta et al. (2021) involved acoustically powered nanobots for enhanced tumor penetration. These micro/nanorobots could actively swim through dense tumor extracellular matrices, delivering chemotherapeutic agents more effectively than passive nanocarriers, significantly improving treatment efficacy in vitro tumor spheroid models [49].

Beyond drug delivery and diagnostics, nanorobots can actively participate in cancer treatment through various therapeutic interventions. Photothermal and photodynamic therapies utilize nanorobots to generate localized heat or reactive oxygen species upon light activation, selectively destroying cancer cells. Hu et al. demonstrated light-driven nanobots capable of generating reactive oxygen species within tumor tissues for photodynamic therapy, resulting in efficient tumor cell eradication in vitro and in vivo [50]. Nanorobots can also enhance radiotherapy’s effectiveness by increasing tumor cells’ sensitivity to radiation. In gene therapy, nanorobots are efficient vectors for delivering therapeutic genes or RNA interference molecules to cancer cells. In addition, nanorobots show potential in revolutionizing cancer surgery, enabling minimally invasive tumor removal, precise identification of tumor margins, and even microsurgery on blood vessels supplying tumors. These surgical applications, coupled with the ability to monitor post-surgical sites and deliver localized treatments, highlight the versatility of nanorobots in comprehensive cancer care [46].

## Integrations of Al and machine learning with nanobots and nanocarriers

Emerging research on serum-derived exosomes underscores their utility as nanoscale vesicular carriers for oncological applications, offering intrinsic biocompatibility and endogenous targeting capabilities. These extracellular vesicles, ranging from 30 to 150 nm, naturally encapsulate biomolecules such as proteins, lipids, mRNA, and microRNA, facilitating intercellular communication within the tumor microenvironment. Integrating exosome-mimetic features into nanocarrier systems or engineering nanobots capable of interfacing with exosomal pathways can enhance selective tumor targeting, reduce immunogenic responses, and improve therapeutic payload delivery. In addition, AI and Machine Learning-driven analysis of exosome-derived molecular signatures enable precise cancer biomarker identification and support the development of adaptive, intelligent nanodevices for real-time monitoring and personalized monotherapy.

Integrating Artificial Intelligence (AI) and Machine Learning (ML) with nanobots and nanocarriers is becoming increasingly essential to enhance their functionality and efficiency in complex tasks, particularly in medical and environmental applications. Traditional methods of controlling these nanoscale devices are often limited in adapting to dynamic environments or making real-time decisions based on evolving data. AI and ML algorithms can address this challenge by enabling nanobots and nanocarriers to process vast amounts of information, optimize their behavior autonomously, and improve their real-time performance. AI-powered nanobots, for example, can learn from their environment, detect anomalies, and adjust their operations without human intervention, offering precision and adaptability. Similarly, ML algorithms can enhance the targeting capabilities of nanocarriers by analyzing large datasets to identify the most effective routes for drug delivery or cellular interaction, leading to improved therapeutic outcomes. These technologies not only enable smarter, more efficient designs but also pave the way for personalized medicine, where treatments can be tailored based on individual patient data**.** Artificial Intelligence (AI) advancements are revolutionizing the medical field, particularly through the emergence of nanobots. These tiny robotic agents can potentially revolutionize drug delivery by precisely targeting therapeutic substances to diseased cells. This innovation could significantly improve cancer patient care by reducing side effects and improving treatment outcomes [47]. The concept of technological singularity speculates about a future where machines surpass human cognitive abilities, marking a major technological shift [48].

AI-integrated nanobots are sophisticated software entities capable of detecting entity status, understanding the ontology of objects, conducting behavioral analysis, learning from experience, and employing cognitive computing intelligence. AI nanobots represent highly intelligent software systems poised towards the futuristic concept of singularity. Artificial Intelligence optimally utilizes the properties of materials to predict nano-interactions with biological fluids, immune system vasculature, targeted drug deliveries, and cell membranes, all of which significantly influence cancer treatment efficacy [48]. Efficient delivery of anticancer medications into the body’s tissues is crucial to ensure they reach every cancer cell within the target population at a concentration sufficient to exert therapeutic effects. While much research on cancer chemotherapy resistance has focused on molecular factors, the importance of restricted drug distribution within tumors has often been overlooked. Consequently, nanobots are gaining prominence for their ability to execute supervised functions at the nanoscale, offering promising solutions to enhance drug delivery efficacy and combat chemotherapy resistance [49].

AI-driven nanobots extensively utilize Deep Learning (DL) and Machine Learning (ML) techniques to execute pharmacokinetic prediction. Chou et al. devised a physiologically-based pharmacokinetic (PBPK) model by integrating an AI-based quantitative structure–activity relationship (QSAR) model with a PBPK model to forecast the tumor-targeted delivery efficiency (DE) and biodistribution of various nanoparticles (NPs). They used ML and deep neural network methods to anticipate crucial input parameters for the PBPK model within the development of the AI-based QSAR model [50]. Significant progress in nanomedicine-based cancer diagnosis and treatment technologies has enabled early disease detection and enhanced medication efficacy while minimizing adverse effects.

Loibl and co-workers reported an Artificial Intelligent Label-Free SERS profiling approach of serum exosomes to diagnose and evaluate breast cancer postoperatively [51]. Although exosomes are naturally derived, their nanoscale dimensions and biological cargo-carrying capability resemble the functional principles of synthetic nanocarriers. Their study, utilizing a supervised ANN algorithm to analyze SERS properties, underscores how AI-driven methods can process complex nanoscale datasets—a strategy increasingly adopted in evaluating the targeting efficiency and therapeutic performance of nanobots and nanocarriers in cancer diagnostics and therapy. Similarly, Hsieh et al. demonstrated ANN-based early breast cancer diagnosis, reinforcing the importance of ML in nanoscale biomedical applications. These AI-integrated approaches are critical in optimizing and monitoring nano-drug delivery systems, enhancing their precision, safety, and therapeutic outcomes in oncology [52, 53].

Computational models, which rely on algorithms, are expected to improve their predictive efficacy for nanomedicines by establishing extensive databases containing nanomedicine libraries and associated physicochemical properties, functional evaluations, and i*n vivo* delivery data. This development raises questions about the application of AI in life-saving efforts for patients, particularly concerning the high mortality rates observed among individuals diagnosed with cancer due to tumor heterogeneity and delayed identification at earlier stages [54]. Zhang et al. conducted a study utilizing blood profile data as a non-invasive ML technique for early detection of metastases from breast cancer, employing algorithms such as Decision Tree (DT), alongside traditional imaging tests and biopsies. While conventional imaging techniques like CT and MRI benefit from AI algorithms for image-guided treatment and monitoring patient response during treatment, efforts are also underway to integrate AI with nanobot technology for precise cancer treatment across various types of cancer [55]. In addition, bioinformatics research focuses on next-generation sequencing of targeted gene sequences to identify complex biomarkers associated with different cancer types. Developing reliable and reproducible bioinformatics tools is crucial for extracting molecular characteristics of each patient’s tumor from NGS data in this context [56].

Artificial Intelligence (AI) has revolutionized micro/nanorobots (MNRs), offering significant advantages such as material synthesis, optimal design, fabrication, and swarm behavior [56]. Machine Learning is undergoing continuous technical advancements to empower tiny nanobots to learn from training datasets and predict cancer types. Wu et al. demonstrated the classification of triple-negative breast cancer and non-triple-negative breast cancer using the Support Vector Machine (SVM) algorithm applied to gene expression datasets. Table 2 illustrates the integration of various AI models with nanobots currently employed in cancer treatment.

**Table 2:** Different types of AI models integration with nanobots for cancer therapy

Models	Applications	Types of cancer	References
Reinforcement Learning	Controlling the drug release mechanisms, cancer diagnosis	Brain tumors, prostate cancer, skin lesions, breast cancer	[57]
Genetic algorithms	Chemo drug formulation, nanobot designing	Pancreatic cancer, colon cancer, cervical cancer, lung cancer	[55]
Evolutionary algorithms	Design of nanobots, drug delivery efficiency, nanobot bearded drug biocompatibility	Lung cancer, breast cancer	[54]
Swarm intelligence algorithms	Coordinate the function of several Nanobots within the body. Lessen the side effects of healthy cells while killing malignant cells	Prostate cancer, breast cancer,	[58]
Deep learning algorithms	Deep Learning algorithms, especially recurrent neural networks (RNNs) and convolutional neural networks (CNNs), are used to analyze imaging data like MRI and CT scans and navigation in nanobots	Breast cancer, lung cancer, oral cancer, breast cancer	[59]

## Active drug delivery through nanobot

**Fig. 6:**
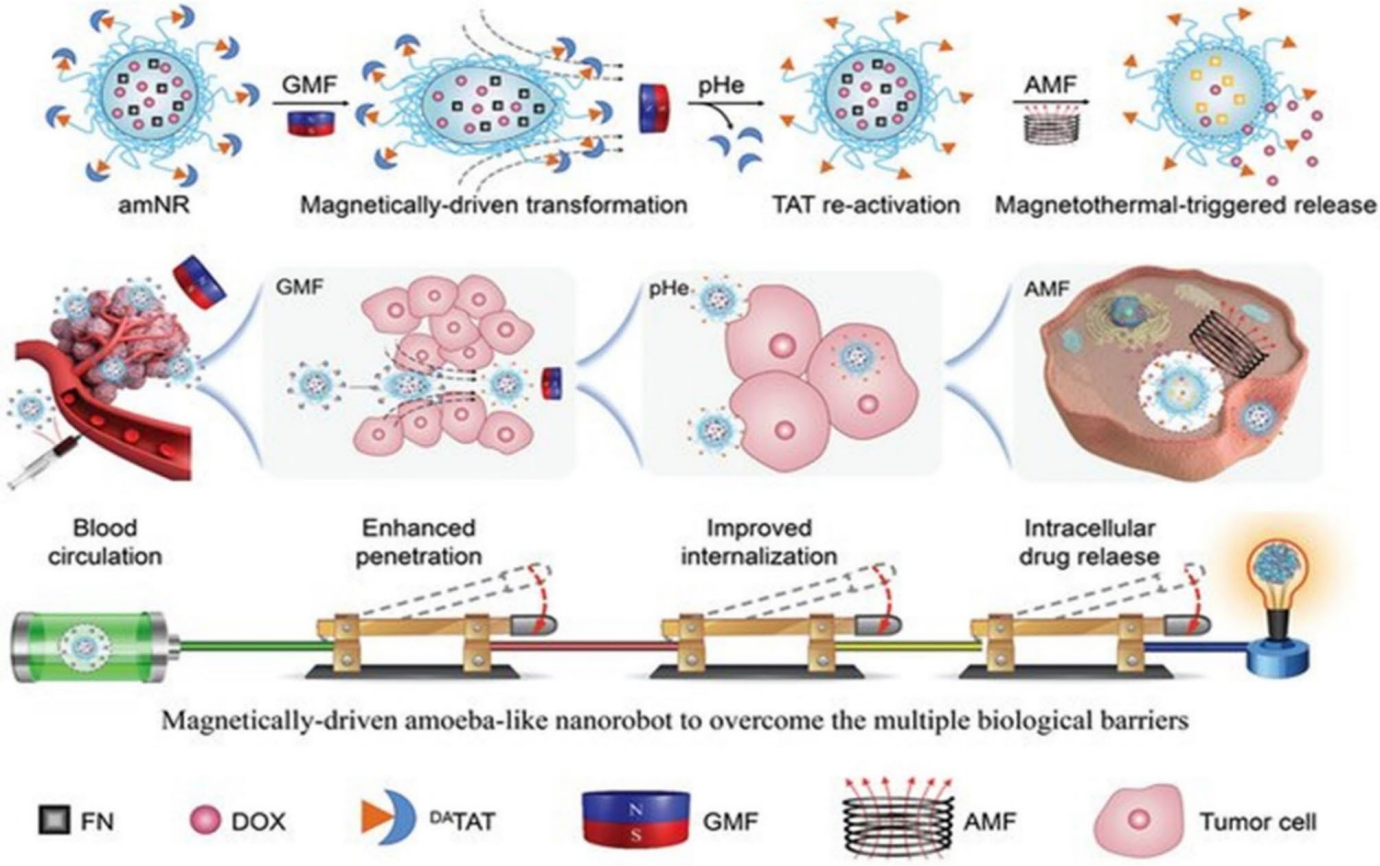
Diagram shows how amNR’s structure changes when applying a magnetic field [62]

Active drug delivery to tumor cells is often hindered by challenges such as poor tumor penetration, insufficient accumulation, rapid blood clearance, and susceptibility to phagocytosis [60]. Recently, there has been growing interest in nanobots with shape-changing capabilities for addressing these limitations. Inspired by the adaptive conformational abilities of amoebas, researchers have developed a magnetically propelled nanobot called amNR (Fig. 6). Constructed with a polyphosphoester (PPE) core, amNR exhibits a deformable structure, allowing it to change shape under magnetic actuation [54]. This core material possesses a low glass transition temperature, facilitating the nanobot’s flexibility when subjected to a magnetic field. Within the core, amNR encapsulates the anticancer drug doxorubicin (Dox) and ferrimagnetic nanocubes (FN) for magnetic manipulation. Upon exposure to a guiding magnetic field (GMF), amNR undergoes distortion, enabling aggressive extravasation into deep tumor tissues. As it penetrates further, the tumor’s acidic environment triggers the exposure of hidden ligands, such as the transactivator of transcription (TAT) peptide, facilitating active uptake by tumor cells [54]. Subsequently, applying an alternating magnetic field (AMF) induces intracellular Dox release from the amNRs. By manipulating magnetic fields, these deformable nanobots achieve comprehensive active drug transport, ultimately enhancing the efficacy of anti-tumor treatments [61].

**Fig. 7:**
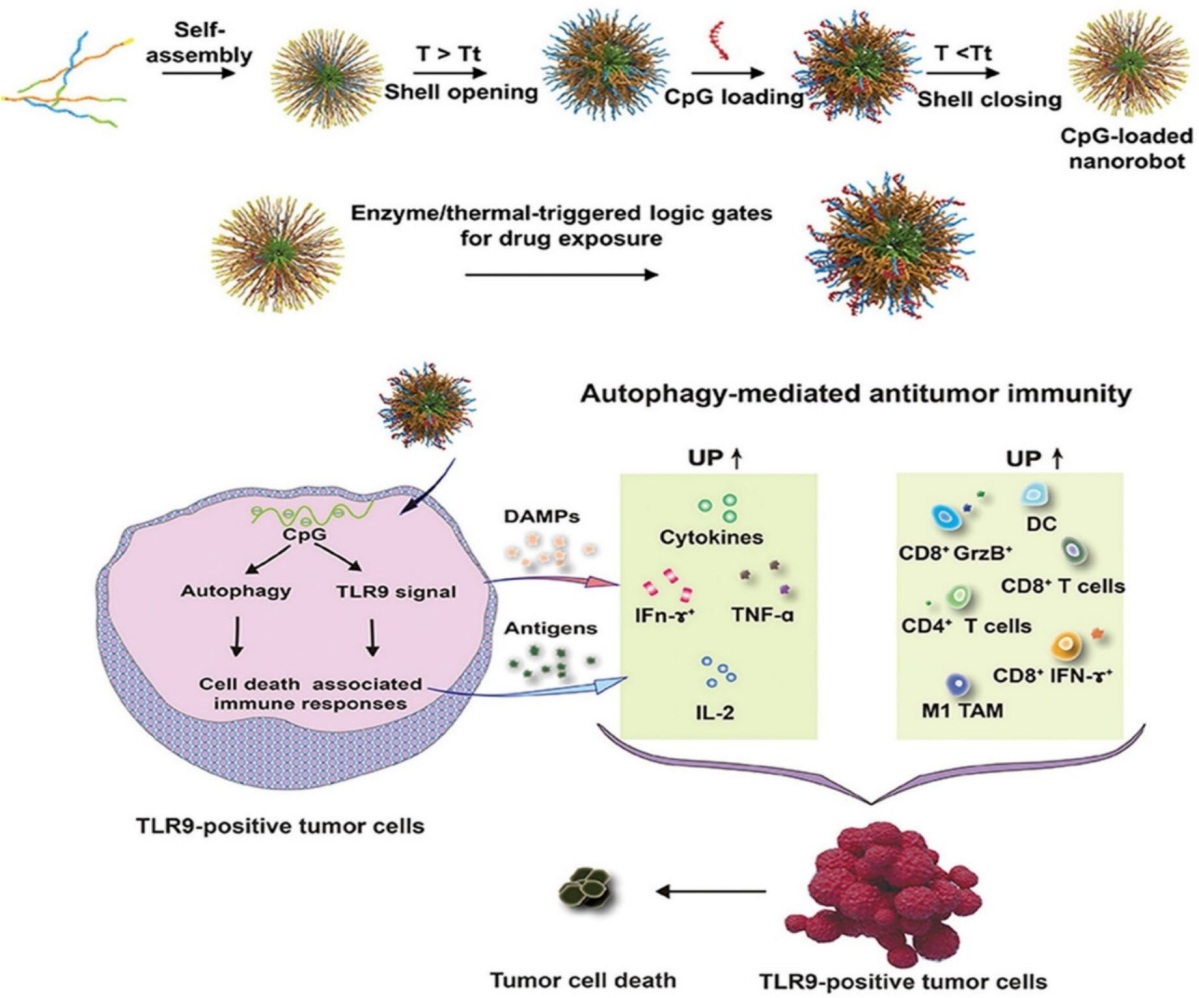
**S**ynthesis of enzyme-activated CpG‐loaded nanobots [67]

Wang et al. engineered a nanobot designed for delivering CpG injections. This nanobot utilized a co-self-assembly approach for fabrication, incorporating two types of amphiphilic triblock polymer peptides [63]. One peptide contained a GRRRDRGRS sequence rich in arginine, facilitating nucleic acid payloads’ condensation through electrostatic interactions. The other peptide contained a matrix metallopeptidase 2 (MMP2)-cleaved GPLGVRGS sequence, enabling the controlled opening of the nanobots. The enzyme-activated CpG-loaded nanobot was synthesized (Fig. 7) by self-assembling these triblock polymer peptides [64]. Nucleic acid drugs were encapsulated within positively charged peptides, and the nanobots’ shells could be opened at elevated temperatures. This strategy allowed for the controlled release of payloads in response to specific enzymatic cues [65]. Nanorobots are created by self-assembling two triblock polymer peptides, releasing pharmaceuticals in the presence of the enzyme MMP2. These nanorobots have shells that open at elevated temperatures, exposing positively charged peptides that contain nucleic acid medications [66].

A recent study by Chen et al. introduced a hydrogel nanobot tailored to target HER2/CD44 malignant tumor cells in HER2-positive breast cancer [68]. This nanobot was engineered using liposome-encapsulated nanoparticles (LPR), incorporating components such as the cationic liposome DOPE, pro-apoptotic peptide KLA, survivin siRNA, and chemically crosslinked Herceptin monoclonal antibody. The interaction between DOTAP/DOPE cationic liposome and t KLA-R16 established an electrostatic connection with siRNA, causing the reorganization and collapse of lipids on the surface of KLA-R16/siRNA complexes [69]. Subsequently, a positively charged ternary composite nanoparticle LPR was formed by integrating these modular parts. Chemical crosslinking was employed, potentially modifying Herceptin’s properties or enhancing its efficacy as a cancer treatment. In addition, hyaluronic acid (HA) was utilized to create the nano-delivery tool, resulting in the HER2/CD44-targeted hydrogel nanobot, ALPR, effectively delivered to HER2/CD44 expressing tumor cells [70].

**Fig. 8:**
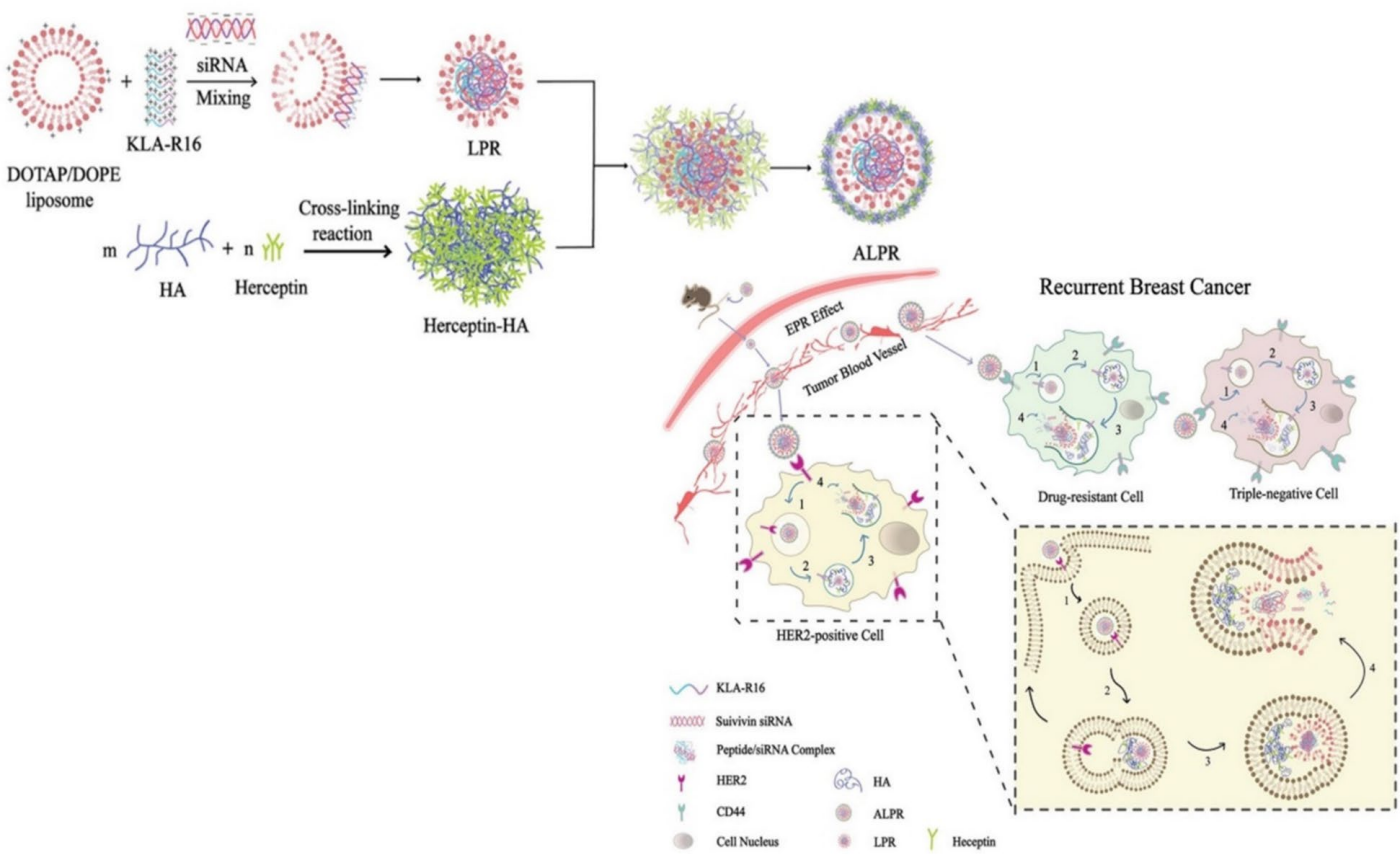
A schematic depicting the process of preparing ALPR by integrating LPR with Herceptin-HA and their characterization and in vivo anticancer efficaciousness of ALPR [70]

Survivin, a protein produced by the BIRC5 gene, is pivotal in impeding apoptosis and is often found in elevated levels in cancer cells, where it impedes programmed cell death [71]. Nevertheless, introducing survivin siRNA can effectively diminish survivin gene expression at the mRNA level in tumor cells. The effectiveness of the ALPR nanobot (Fig. 8) was assessed against NIH3T3, SKBR-3, and MDA-MB-231 cells, displaying notable anticancer properties. Notably, experiments on xenograft tumors originating from MDA-MB-231 and SKBR-3 cells revealed ALPR’s efficacy in restraining tumor growth. In contrast, individual administrations of free survivin siRNA, KLA-R16, and Herceptin-HA had negligible impact on survivin gene expression within tumor cells. Conversely, ALPR displayed the capability to reduce survivin expression, thereby encouraging apoptosis [72]. Furthermore, ALPR effectively halted the proliferation of malignant breast tumor cells through mitochondrial interference, survivin gene suppression, and inhibition of HER2 receptors on the surface of HER2-positive cells [73].

**Fig. 9:**
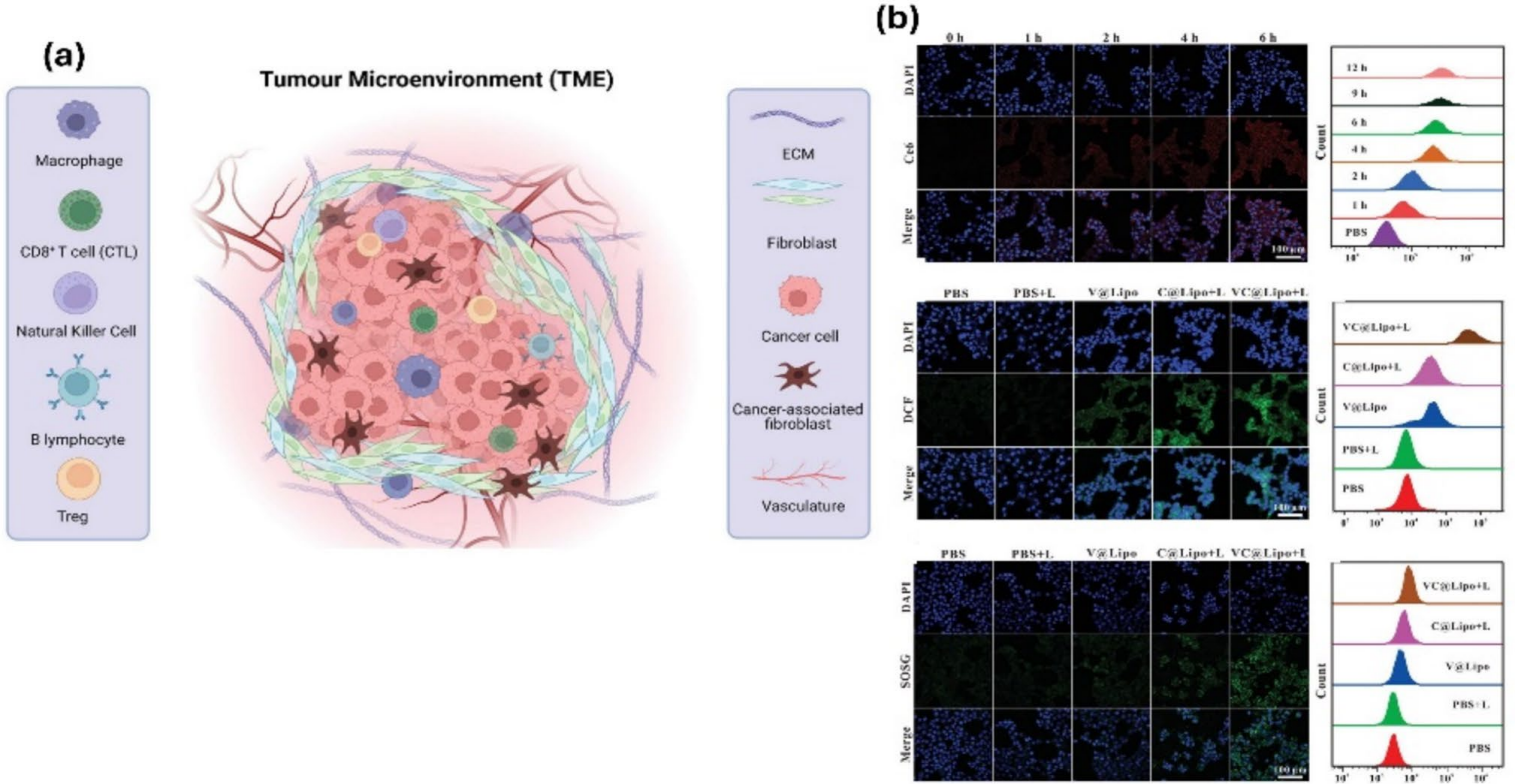
**a** Illustration of TME, encircling tumors, consists of immune and nonimmune cell types, extracellular matrix, and acidic pH. NK and CTLs suppress tumor growth, while Tregs encourage tumor spread. **b** The tumor cell’s absorption of the nanocarrier and the creation of intracellular ROS. Fluorescence imaging and quantitative measurement of intracellular Ce6 fluorescence in 4T1 cells incubated with VC@Lipo at varying periods using a flow cytometer; fluorescence imaging, and flow analysis of intracellular ROS by DCFH-DA, ^3^O2 by SOSG in 4T1 cells treated with varying doses [76]

In recent years, with advancements in cancer research focusing on diagnosis and treatment, there has been significant interest in understanding the ecosystem surrounding oncogenic cells, known as the tumor microenvironment (TME) (Fig. 9). This encompasses various non-cancerous components such as circulating immune cells, signaling molecules, fibroblasts, and blood vessels. The TME plays a critical role in tumor pathogenesis, as it provides a conducive environment for cancer cells to thrive and interact, often through the lymphatic and circulatory systems, leading to tumorigenesis [74]. TME is characterized by elevated levels of glutathione (GSH), H_2_O_2_, and decreased pH concentration, contributing to cancer persistence through intricate cell–cell interactions (CCIs**)** [75]. Tumor cells depend on an antioxidant system centered around GSH to combat oxidative stress and enhance their resistance to reactive oxygen species (ROS) [76].

Addressing this challenge, Zhang et al. proposed a novel anti-tumor strategy involving a Vox nanozyme-based nanocarrier for tumor therapy. This nanocarrier encapsulated vanadium-based nanozyme Vox and the photosensitizer chlorin e6 (Ce6) within liposomal nanovesicles, exhibiting peroxidase activity in weakly acidic conditions [77]. Ce6 induces apoptosis through caspase-3 activation and generates high levels of ROS due to its photosensitizer properties. By leveraging Vox nanozyme, photodynamic therapy (PTD) produces cytotoxic ROS, depleting GSH through redox reactions and impairing its antioxidant function within the TME. This approach effectively inhibits tumor cells by disrupting their antioxidant defenses, ultimately enhancing therapeutic outcomes.

**Fig. 10:**
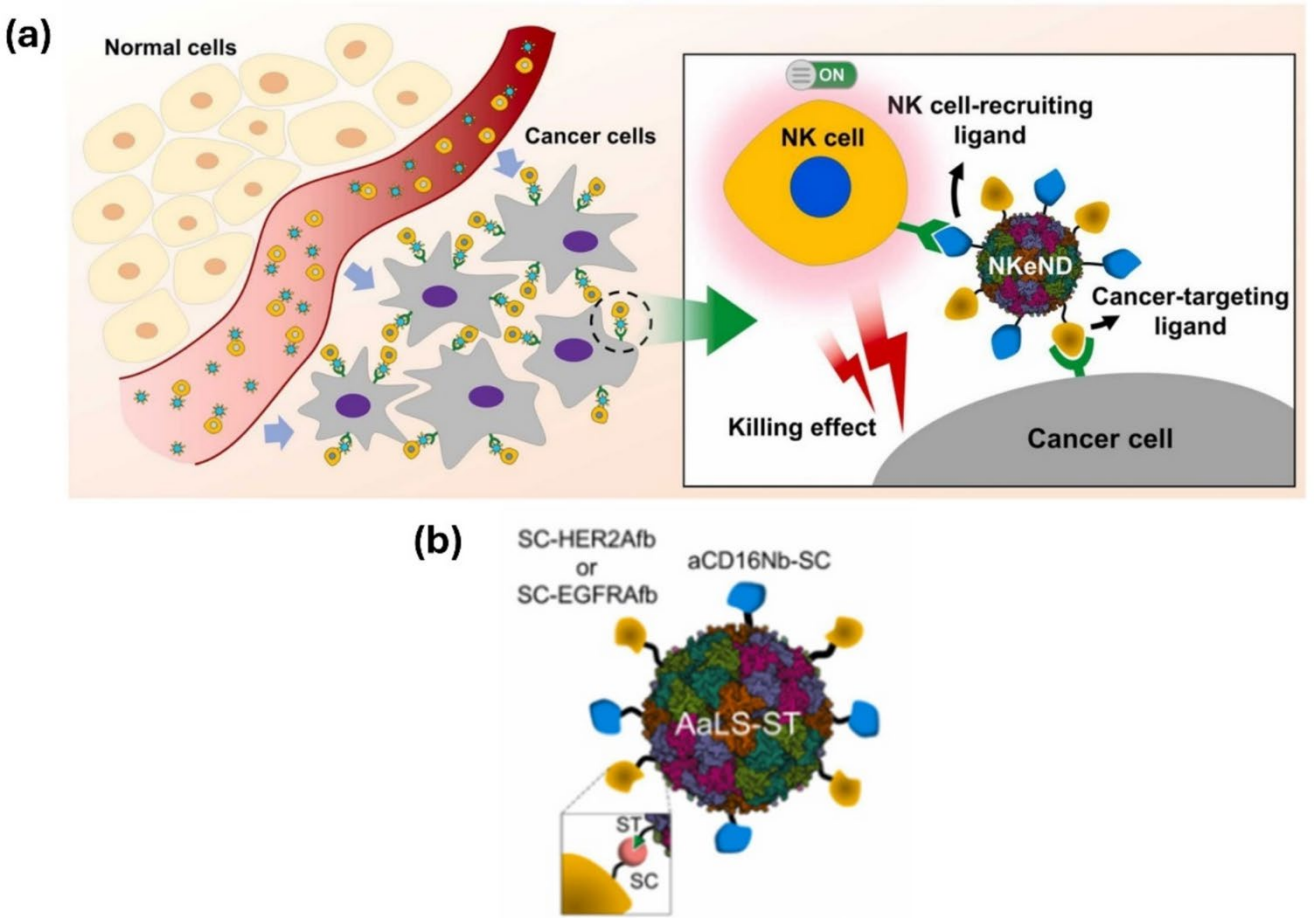
**a** Illustration showing NK cell-engaging nanodrones (NKeNDs) based on protein cage nanoparticles efficiently attract NK cells to specific tumor locations and inhibit tumor development. **b** An example of NKeND schematically. SDS-PAGE examination of the resulting proteins that were post-translationally ligated between AaLS-ST and SC-HER2Afb and/or aCD16Nb-SC or SC-EGFRAfb and/or aCD16Nb-SC [79]

Park et al. recently conducted a study demonstrating the use of nanodrones for cancer immunotherapy, where bioengineered nanodrones interacted with natural killer (NK) cells to enhance tumor-targeting efficiency. Unlike conventional nanocarriers, nanodrones exhibit specialized immune-modulating functions, making them a promising tool for cancer treatment. NK cells are cytotoxic cells (Fig. 10) that eliminate targeted cells by releasing granules containing granzymes, perforin, and cytokines that engage other immune cells against tumor cells in the tumor microenvironment. Engager proteins facilitate the interaction between NK cells and targeted cancer cells [78]. In contrast to traditional methods, nanoparticles have been developed to enhance NK cell function. These nanoparticles can bind multiple ligands to the surface of NK cells, improving tumor penetration, extending blood circulation, and enhancing retention time for nanodrone-assisted NK cells [79].

## Challenges in large utilization of nanobots and nanocarriers in cancer treatment

The clinical translation of nanobots and nanocarriers faces several challenges despite their immense potential to revolutionize medical treatments [80]. One major hurdle is ensuring the safety and biocompatibility of these nanomaterials within the complex biological milieu of the human body. Nanoparticles must not induce cytotoxicity, immunogenicity, or other adverse effects that could compromise patient health.

Moreover, achieving precise targeting and controlled release of therapeutic payloads remains a formidable task. While nanobots offer the advantage of targeted delivery, ensuring their precise navigation to diseased tissues while avoiding off-target effects is a considerable challenge [81]. Administration routes and designs of nanobots for different types of cancers are mentioned in Table 3.

**Table 3:** Administration routes and designs of nanobots for different types of cancers

Route of administration	Type of cancer	Design and synthesis of nanobot	References
Inhalation	Lung cancer	Lipid-based nanobots deliver chemotherapy using liposomes or lipid nanoparticles for lung cancer	[82]
		Gold nanobots use gold nanoparticles to target and detect cancer cells precisely	
Topical application and intravenous injection	Skin cancer (melanoma)	Peptide-based nanobots target melanoma cells using peptides for binding	[83]
		Thermo-sensitive nanobots release drugs specifically at the tumor site, triggered by laser treatment	
Intravenous Injection	Breast cancer	Polymer-based nanobots use biocompatible polymers like PLGA to encapsulate drugs for targeted delivery	[84]
		DNA origami nanobots transport drugs and target cancer cells by folding DNA strands into specific shapes	
Intertumoral injection and intrathecal injection	Brain tumor (glioblastoma)	Dendrimer-based nanobots deliver drugs across the blood–brain barrier	[85]
		Magnetic nanobots use magnetic properties that are guided by external magnetic fields for targeted delivery to brain tumors	
Transrectal injection and intravenous injection	Prostate cancer	Hybrid nanobots combine inorganic and organic elements for versatile therapy and imaging applications	[86]
		Cell-derived nanobots enhance targeting and reduce immune response by encapsulating nanoparticles in cell membranes	
Endoscopic ultrasound-guided injection and intravenous injection	Pancreatic cancer	RNA-based nanobots use RNA nanoparticles for gene regulation and targeted drug delivery	[87]
		Polymeric micelles encapsulate chemotherapy drugs and deliver them to pancreatic cancer cells via self-assembling nanocarriers	
Intravenous injection and intrathecal injection	Leukemia	Magnetic iron-oxide nanobots use magnetic properties for targeted delivery to leukemia cells	[88]
		Biodegradable nanobots decompose after releasing their payload to reduce systemic exposure	

In addition, the scalability and reproducibility of nanomanufacturing processes pose obstacles to large-scale production for clinical applications. Regulatory approval also presents a significant barrier, as stringent standards must be met to ensure the safety and efficacy of nanotherapeutics. Furthermore, the long-term fate and potential accumulation of nanoparticles in the body and their environmental impact necessitate thorough investigation and risk assessment. Overcoming these challenges requires interdisciplinary collaboration among scientists, engineers, clinicians, and regulatory agencies to address safety concerns, optimize design strategies, and navigate regulatory pathways, ultimately facilitating the successful translation of nanobots and nanocarriers into clinical practice [89].

### Physiological barriers

Nanobots and nanocarriers must navigate the bloodstream to reach tumor sites, which is crucial for their effectiveness in vivo. However, the rapid clearance of drugs from the bloodstream within a short time frame can hinder their efficacy. Prolonged circulation of nanoparticles may lead to their accumulation in various organs and tissues, potentially resulting in toxicity or adverse side effects. Moreover, turbulence, shear stress, and high blood flow rates can obstruct their path or cause damage before reaching the target [90]. One major physiological obstacle is the blood–brain barrier (BBB), a selective membrane that tightly controls the interaction between blood vessels and the central nervous system. This barrier restricts the entry of therapeutic drugs and impedes the delivery of nanobots, nanocarriers, and their payloads, making effective treatment more difficult [91].

Once nanobots and nanocarriers exit the bloodstream and reach the tumor site, they face additional pharmacokinetic challenges within the tumor microenvironment (TME). The TME is a hostile and unique environment characterized by low oxygen levels (hypoxia), acidic pH, and high interstitial fluid pressure, all of which can negatively affect the behavior and performance of nanobots [92]. In addition, cancer cells often evade immune responses, primarily due to the high lactic acid production and acidic conditions in the TME that suppress various immune cells. Furthermore, the ability of nanobots and nanocarriers to enter tumor cells may be limited by cellular membranes. However, this challenge can be mitigated by modifying the nanoparticle surface or designing nanocarriers and nanobots that mimic cellular membranes [93]. For example, Hu et al. (2015) developed cancer cell membrane-coated nanoparticles (CCNPs) capable of evading immune recognition and improving tumor targeting by mimicking the surface antigens of cancer cells, which facilitated homotypic targeting and enhanced cellular uptake [93]. Similarly, Harris et al. demonstrated that coating nanoparticles with red blood cell (RBC) membranes prolonged their circulation time and reduced macrophage clearance, enabling the carriers to remain in systemic circulation longer and enhancing their tumor accumulation efficiency [94]. Another innovative approach by Luk et al. involved leukocyte membrane-coated nanocarriers, which exploited the natural homing ability of immune cells to inflamed or cancerous tissues, improving targeted delivery in the tumor microenvironment [95]. These bioinspired strategies not only improve biocompatibility and reduce immunogenicity but also enhance the ability of nanobots and nanocarriers to penetrate cellular membranes and deliver therapeutic payloads directly into tumor cells.

### Targeting specificity

Examining the hurdles associated with precisely targeting diseased cells or tissues while mitigating off-target effects is crucial in drug delivery systems (DDS) [94]. Conventional delivery methods often fail to achieve sufficient penetration and targeting, leading to reliance on bodily fluids circulation for medication distribution and inadequate access to certain areas like the interior of cancerous cells [95]. Nanocarriers and nanobots offer promising solutions by leveraging various energy sources for efficient motion, thus revolutionizing targeted medication administration. Drugs can be directed to specific bodily regions or tissues through targeted transport and triggered release mechanisms. Various energy sources, including self-propelled microorganisms such as bacteria, sperm, and immune and contractile cells, as well as external field-propelled nanobots and nanocarriers like light, electrical, acoustic, and magnetic fields, are considered for targeted transport [96].

In addition, achieving precise intracellular delivery poses a significant challenge. Nanocarriers encounter multiple cellular barriers, including endosomal entrapment and lysosomal degradation, which hinder the delivery of therapeutic cargo to specific subcellular compartments. For instance, Feng et al. conducted a study where they synthesized iR@ZIF-8 nanocomposites, utilizing nanoscale zeolitic imidazolate framework (ZIF) as a vector for miRNA delivery, effectively evading endosomal entrapment and lysosomal degradation. Moreover, nanobots can be chemically conjugated with nanoparticles like magnetic Fe_3_O_4_ to facilitate self-propulsion and targeted penetration. However, self-propelling nanobots may encounter challenges of uncontrolled motion. Hence, nanobots and nanocarriers designed using swarm strategies must incorporate appropriate propulsion mechanisms and control systems to navigate diverse physiological environments. Furthermore, nanobots and nanocarriers need a stable and reliable power source to sustain their propulsion and functionality within the body.

### Cost and expense

The financial impact of cancer is a growing global concern, encompassing not only direct medical expenses but also secondary costs such as missed productivity, caretaker stress, and the emotional toll on patients and families [9]. Studies have shown significant financial burdens on individuals, with a considerable portion of breast cancer patients reporting yearly health expenses exceeding ten percent of their income [3]. Furthermore, the frequency of cancer cases is expected to rise, affecting a substantial proportion of the population in countries like the UK [2]. However, healthcare systems, such as the UK National Health Service, are grappling with challenges such as severe staffing shortages and delays in cancer care, exacerbated by the COVID-19 pandemic [13].

In response, new therapies are being developed, with extensive research on nanocarriers and nanobots transitioning from in vitro to in vivo studies and from laboratory settings to the pharmaceutical market [21]. Nanocarriers have made significant strides in increasing the bioavailability of poorly soluble medications and improving access to pathological sites [10]. However, despite their potential, nanobots still face limitations in terms of cost affordability for widespread clinical usage. Factors such as research and development costs, manufacturing expenses, regulatory compliance, and clinical application contribute to the high costs associated with nanobot development and deployment in cancer therapies [8].

### Biocompatibility and toxicity concerns

Various research studies have extensively investigated the toxicity profile of nanobots and nanocarriers designed for cancer therapy. Issues such as drug resistance, adverse effects, and suboptimal efficacy have hindered the progression of clinical studies in cancer precision medicine. Due to their nano-size and function of loading and releasing drugs in living systems, the immune system may perceive nanobots and carriers as foreign agents, potentially triggering immune responses. However, advancements in bioinspired design have led to the development of biocompatible nanobots. For instance, biohybrid cell-based nanobots have garnered attention due to their biocompatible and biodegradable properties. Researchers like Chen and his team [95] have explored the potential of bioinspired designs in biomedical applications. In one such attempt, Xing et al. engineered bioinspired jellyfish-like carbon/manganese nanomotors with dual-propulsion capabilities using H_2_O_2_ and NIR light. This innovative design mimics the natural movement of jellyfish, resulting in enhanced tumor penetration and chemodynamic therapy. Overall, bioinspired design strategies promise to mitigate the toxicity concerns associated with nanobots and nanocarriers in cancer therapy.

As demonstrated by Zhou and colleagues [69], a biocompatible nanocarrier has been developed for delivering Dox and siRNA, utilizing nanocarriers derived from α-lactalbumin nanoparticles obtained from food. While the immune system may attack administered nanobots and nanocarriers, strategies such as nanoengineering surfaces or cellular membranes, as shown by Song et al., can help evade or manage such immune responses. In addition, nanorobots or nanocarriers have the potential to reprogram tumor immunosuppressive phenomena, thus directing the body’s immune system to attack and destroy cancerous cells. Biohybrid nanostructures show promise for immunosuppression, with examples including enzyme-conjugated magnetic nanoparticles designed to target human breast cancer cells by mimicking the innate immune cell’s respiratory explosion response. Moreover, nanocarriers derived from yeast have demonstrated biocompatibility against cancer, serving as effective delivery agents in living systems. Table 4 provides a summary comparison of the respective levels of efficiency of several nanobots and carriers.

**Table 4:** Comparison efficiency between different nanobots and nanocarriers

Efficiency	Nanobots	Nanocarriers	References
Targeting and specificity	Active targeting:	Passive targeting mechanisms	[97]
	Remote control:	Vascular permeability	
	Responsive to stimuli:	Tumor heterogeneity	
		Limited penetration	
		Response to stimuli	
Design and formulation	Advanced design	Maximizing encapsulation efficiency	[98]
	Versatility	Particle engineering techniques	
	Integrated components	Controlled release mechanisms	
	Sophisticated engineering	Surface modifications	
Navigation	Active navigation	Controlled release	[99]
	Adaptability	Enhanced bioavailability	
	Autonomous traversal	Improved therapeutic outcomes	
		Increased treatment efficiency	
Payload and multi-functionality	Versatile multitaskers	Versatile encapsulation	[100]
	Drug loading	Enhanced treatment efficiency	
	Medication transport	Diverse therapeutics delivery	
	Imaging and sensing		
	Therapeutic intervention		
Biodegradability and clearance	Long-term toxicity concerns	Biocompatible materials	[28]
	Biodegradability issues	Improved biodegradability	
	Clearance mechanisms	Enhanced clearance properties	
	Disposal challenges		

## Conclusions and future perspectives

In this review, we have tried to capture recent updates on nanocarriers and nanobots in the context of cancer treatment. Nanocarriers and nanobots offer precise, efficient, and targeted delivery of therapeutic agents to cancerous cells, while minimizing systemic toxicity and side effects. Researchers have developed sophisticated nanocarrier design and production techniques, enabling the encapsulation of various therapeutic agents such as chemotherapeutic drugs, peptides, nucleic acids, and imaging probes into nano-sized carriers. These nanocarriers enhance solubility, stability, and delivery to tumor sites, with stimuli-responsive materials further improving therapeutic effectiveness. Nanobots play a revolutionary role in cancer treatment by detecting malignant growths, penetrating biological barriers, and administering therapeutic agents with remarkable accuracy.

The integration of AI has significantly improved the use of nanocarriers and nanobots by enabling real-time imaging and feedback systems. AI algorithms can use extensive patient data to personalize treatment plans and forecast response outcomes, optimizing therapeutic effectiveness and minimizing adverse effects. These systems allow for the customization of therapy techniques in real-time, optimizing therapeutic outcomes while reducing off-target consequences. A recent innovative concept involves nanodrones, which can infiltrate tumor tissues, surpassing physiological barriers such as the extracellular matrix and blood vessels, to deliver therapeutic compounds, unlike conventional methods. Nanocarriers and nanobots demonstrate promising therapeutic outcomes across various cancer types, with preclinical studies indicating improved treatment effectiveness and reduced toxicity. However, scalability, manufacturing repeatability, and regulatory hurdles must be addressed for widespread clinical use.

Looking forward, the future outlook for nanocarriers and nanobots in cancer therapy is exceedingly promising. Ongoing advancements in nanotechnology, materials science, and biomedical engineering are poised to improve these platforms’ effectiveness and safety significantly. Researchers are exploring novel approaches to enhance the targeting precision of nanobots and nanocarriers, such as integrating biomarkers or employing stimuli-responsive materials for controlled drug release. For instance, nano-hydrogel-based systems offer advantages like high drug loading capacity, stability, simultaneous targeting, co-delivery of hydrophilic drugs with varying charges, and facilitation of drug release. Furthermore, the evolution of increasingly sophisticated nanobots with enhanced sensing and therapeutic capabilities is expected to enable real-time monitoring of tumor progression and more precise intervention strategies. Furthermore, integrating AI and ML algorithms helps enhance nanocarrier design, prediction of treatment outcomes, and customization of treatment plans based on individual patient profiles.

The fusion of nanocarriers and nanobots with emerging technologies, concurrently gene editing methods such as CRISPR offer the potential to modify nanocarriers with tailored functionalities or bolster the immune-modulating capabilities of nanobots, enhancing their capacity to combat cancer cells. Furthermore, transitioning these innovations from laboratory experimentation to clinical application marks a significant milestone in the battle against cancer. Although challenges persist, encompassing regulatory complexities, scalability limitations, long-term safety considerations, cost factors, and biocompatibility issues, collaborative endeavors among academia, industry, and regulatory bodies are indispensable for surmounting these barriers and expediting the clinical adoption of these breakthroughs.

Ultimately, the widespread adoption of nanocarriers and nanobots in cancer therapy has the potential to usher in a new era of precision medicine. This entails tailoring treatments to the unique molecular characteristics of each patient’s cancer, leading to improved outcomes and enhanced quality of life. The outlook regarding using nanocarriers and nanobots in cancer therapy is notably optimistic, driven by continual advancements in materials science, biomedical engineering, and nanotechnology. Moreover, the development of multifunctional nanoplatforms capable of imaging, diagnosis, and treatment simultaneously holds promise for the emergence of theragnostic medicine, where real-time treatment plans are tailored to the specific genetic traits of individual tumors. In summary, nanobots and nanocarriers represent revolutionary platforms with the potential to transform cancer therapy by providing efficient, personalized treatments, mitigating side effects, and yielding better patient outcomes. This transformation relies on sustained research efforts, interdisciplinary collaborations, and technological breakthroughs.

## Data Availability

The data from this study will be made available on request.
